# P-1742. Isavuconazole Therapeutic Drug Monitoring and Association with Adverse Events

**DOI:** 10.1093/ofid/ofae631.1905

**Published:** 2025-01-29

**Authors:** Emily Huang, Rebecca Wittenberg, Joy Vongspanich Dray, George R Thompson, Jeffrey Fine, Machelle Wilson, Melissa Chee

**Affiliations:** UC Davis Health, Sacramento, California; UC Davis, Sacramento, CA; UC Davis Health, Sacramento, California; University of California Davis Medical Center, Sacramento, CA; University of California, Davis, Davis, California; UC Davis Health, Sacramento, California; Kaiser San Mateo, San Mateo, California

## Abstract

**Background:**

Therapeutic drug monitoring (TDM) is often recommended with triazole antifungals. Isavuconazole TDM is currently not uniformly recommended due to its high oral bioavailability, modest drug-drug interactions, and linear pharmacokinetics; however, an assessment of serum levels and toxicity has not been reported.

This study aimed to explore the exposure-toxicity relationship for isavuconazole. The primary outcome was the threshold isavuconazole serum level most predictive of toxicity. Secondary outcomes included percentage of patients who developed adverse events and identification of adverse event profile.

**Table 1:**
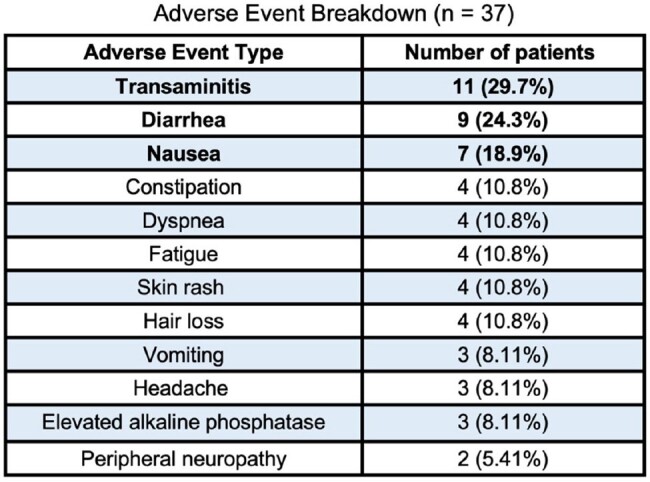
Isavuconazole Adverse Event Breakdown This table details the exact number and percentage of patients who experienced each type of adverse event reported by the patient sample with isavuconazole

**Methods:**

This retrospective study analyzed adult patients who received oral isavuconazole and collected isavuconazole serum levels at our institution from 1/1/2015 to 10/4/2023. T-test and Fischer’s exact test were used to compare patients with and without adverse events, while Youdin’s index was used to identify a serum level most predictive of toxicity.

**Results:**

Ninety-five patients, corresponding to 219 serum levels total (UPLC/MS), were analyzed. Thirty-seven patients (38.9%) developed adverse events, most commonly transaminitis (29.7%), diarrhea (24.3%), and nausea (18.9%) (Table 1). According to Youdin’s index, a serum level of 5.86 mcg/mL corresponded to a threshold that balances sensitivity and specificity for determining the risk of adverse events. Per multivariable logistic regression analysis, each one-unit increase (mcg/mL) in serum level was associated with a significantly increased odds risk of developing toxicity by 26%. All 24 patients who had isavuconazole dose-reduction secondary to adverse events demonstrated resolution of symptoms. Fifteen out of those 24 patients rechecked serum levels following dose reduction, yielding a mean serum level of 3.3 mcg/mL post-reduction, compared to 6.3 mcg/mL pre-reduction.

**Conclusion:**

Our findings identify an exposure-toxicity relationship for isavuconazole. TDM may be beneficial for those on long-term isavuconazole therapy, especially in those who develop toxicity. Additionally, in patients with serum levels above 5.86 mcg/mL who show intolerable symptoms consistent with possible adverse drug effects, dose reduction is recommended.

**Disclosures:**

**George R. Thompson, III, MD**, Astellas: Advisor/Consultant|Cidara: Advisor/Consultant|Cidara: Grant/Research Support|F2G: Advisor/Consultant|F2G: Grant/Research Support|Melinta: Advisor/Consultant|Melinta: Grant/Research Support|Mundipharma: Advisor/Consultant|Mundipharma: Grant/Research Support|Pfizer: Advisor/Consultant

